# HIV and/or AIDS awareness among adolescents in a South African at-risk rural community

**DOI:** 10.4102/sajhivmed.v17i1.418

**Published:** 2016-06-09

**Authors:** Simon Taukeni, Ronel Ferreira

**Affiliations:** 1 HIV and AIDS Unit, University of Fort Hare, South Africa; 2 Department of Educational Psychology, University of Pretoria, South Africa

## Abstract

**Background:**

The devastating effects of HIV and/or AIDS are widely documented. Despite ongoing efforts to address the challenges associated with the pandemic, the impact on children orphaned because of the disease, as well as on adolescents, remains problematic. More specifically, orphaned adolescents living in poverty are particularly vulnerable and are often exposed to, for example, emotional and physical abuse and transactional sexual exploitation. Against this background, the importance of informed awareness among adolescents is continually emphasised, yet the outcomes of awareness campaigns require ongoing research.

**Objectives:**

The main objective of this study was to explore HIV and/or AIDS awareness among adolescents living in a rural community in South Africa, in the Chris Hani District of the Eastern Cape Province. Sixteen adolescents (aged 12–24) who had lost one or both of their parents because of HIV and/or AIDS-related reasons were purposefully selected to participate in the study.

**Method:**

For this qualitative investigation, we implemented a descriptive case study design. Semi-structured individual interviews, observation and field notes were used to collect and document data, and inductive thematic analysis was completed using the software programme Atlas.ti 7.

**Results:**

The three themes that were identified relate to HIV and/or AIDS awareness, disclosure of parents’ HIV and/or AIDS status and experiences of adolescents surrounding the death of their parents. Adolescents of the community viewed HIV and/or AIDS as an infectious disease that can lead to death; however, this can be prevented by avoiding at-risk sexual behaviour. Schools and family members were the main sources of information regarding HIV and/or AIDS to the participants. Even though parents tended not to disclose their HIV and/or AIDS status, adolescents became aware of their parents’ status when reading about this on their parents’ medical report cards or when being told about the status by others following the death of their parents. For adolescents, their parents’ deaths were associated with the parents being chronically ill or showing visible signs of deterioration such as weight loss.

**Conclusion:**

The study concludes that even though current campaigns and informative interventions have seemingly succeeded in ensuring HIV and/or AIDS awareness among adolescents – also those in remote areas – continued educational campaigns are important. Such initiatives may prove to be beneficial by focusing on ways that parents can discuss HIV and/or AIDS-related issues with their children and disclose an HIV-positive status.

## Introduction

UNAIDS^1^ estimates that about 35 million people were living with HIV and/or AIDS in 2013, of which 70.6% were situated in sub-Saharan Africa. In South Africa alone, 2.3 million children had been orphaned by AIDS at the time and around 6.3 million people were living with HIV and/or AIDS.^2^ Worldwide, many of those affected are women and children.

When narrowing the focus to adolescent children and young adults, worldwide figures indicated 10.3 million HIV-positive youth within the age range 15–24.^3^ In many parts of the globe, young people, particularly adolescents, are at great risk of contracting the virus through sexual activity as well as sexual exploitation and abuse.^4^ Durojaiye^5^ as well as UNAIDS^6^ indicate that, worldwide, 45% of people contracting HIV and/or AIDS are youth aged 15–24 years. This is supported by Ebeniro^7^ and other scholars who found that adolescents aged 15–24 years constitute the largest population of HIV-infected subjects. Similarly, AVERT^2^ indicates a high HIV prevalence in the age group 15–29.

Adolescents find themselves in a developmental phase where they search for an identity and are often negatively influenced by their peers. Adding poverty and parental sickness, or orphanhood, to the challenges already experienced during adolescence will result in high levels of vulnerability. More specifically, orphaned adolescents living in poverty are particularly at risk of emotional and physical abuse and transactional sexual exploitation.^8^

## South African context as backdrop to the study

According to Statistics South Africa,^9^ this country accounts for the highest number of people living with HIV and/or AIDS in the world, currently estimated at 10.2% of the total population. Sinelela et al.^10^ confirm this by very recently stating that:

South Africa is home to the largest concentration of people living with HIV anywhere in the world; of all the HIV-positive people in the world, nearly one fifth live in South Africa. (n.p.)

In terms of the rise in numbers, it is rather concerning to note the increase from 4.09 million people living with HIV and/or AIDS in South Africa in 2002 to 5.51 million in 2014.^9^

The HIV and/or AIDS pandemic has caused unprecedented suffering and social disruption for children and communities throughout sub-Saharan Africa.^11^ Even though all children are vulnerable and at risk of contracting HIV, it was, for example, reported in a study by Mpanju-Shumbusho^12^ that, in sub-Saharan Africa, teenage girls’ HIV infection rate is five times higher than the rate of infection among teenage boys. Against the background of the challenge of HIV and/or AIDS encountered by adolescents, it seems vitally important that their awareness of the risks associated with sexual behaviour, and the importance of applying this knowledge to real-life experience, is made clear.^13^

Adolescents (ages 10–19) find themselves in a phase of physical growth and development accompanied by sexual maturation, which often lead to intimate relationships.^4^ Additionally, adolescents orphaned by HIV and/or AIDS or living with sick parents as a result of the disease, in poor conditions, are specifically vulnerable to transactional sexual exploitation.^8^ Even though the 1998 South African Demographic and Health Survey indicated that awareness and knowledge about HIV and/or AIDS are high among adolescents in South Africa, this has seemingly not translated into substantial behaviour change among adolescents.^14^

However, South Africa has a range of relevant policies and interventions geared towards fighting the HIV and/or AIDS scourge^15^ in place. Examples of these include the National Strategic Plan on HIV and/or AIDS and STD 2000–2005, the National Strategic Plan on HIV and AIDS and STI 2007–2011, and the current policy of National Strategic Plan on HIV, STIs and TB 2012–2016. These policies are an indication that the South African government and its partners have the intention to address the HIV and/or AIDS pandemic, despite attempted actions not yet showing results in terms of changed behaviour and a reduction in HIV and/or AIDS prevalence among this age group.

Social challenges and the current state of affairs in South Africa add to the vulnerability of adolescent children. Currently, 53.8% of all South African citizens are considered to live in poverty,^16^ and the unemployment level is estimated to be 25.5%.^17^ Poverty and high levels of unemployment have resulted in people at the ground level pursuing all possible avenues to survive, even if this may place the well-being of the family and children at risk. It is a common phenomenon that parents seek employment away from home, resulting in a situation where many children reside with relatives or even friends. Such living arrangements and the lack of parental involvement intensify the vulnerability of children, more so during the developmental phase of adolescents, when children are in need of the involvement and support of caring adults.

In addition to the developmental phase and prominent effect of peer pressure adding to the vulnerability of adolescents not cared for by their parents, social challenges such as abuse, violence and promiscuity in poor communities in South Africa add to the challenges these children face. Furthermore, the high prevalence of HIV and/or AIDS and related deaths in South Africa have resulted in high numbers of orphaned children – 1.5 million in 2009 and estimated to increase to almost 1.8 million children in 2015.^18^ These children need to be cared for, or in some cases where parents live with HIV and/or AIDS, children are expected to take over the role of provision from their parents, in order to support sick parents and siblings to survive.^19^ The death of parents and migration to other regions in pursuing careers, have also resulted in the phenomenon of child-headed households in South Africa. This further intensifies the population of vulnerable and at-risk children (often adolescents) in South Africa.

Against this background, the present study explored the awareness of HIV and/or AIDS among adolescents whose parents had died of HIV and/or AIDS, in an at-risk rural community in the Chris Hani District in the Eastern Cape of South Africa. The rationale for undertaking the study rested on scientific evidence indicating that knowledge about HIV and/or AIDS is essential for adolescents to be able to make rational decisions regarding sexual behaviour and protecting themselves against HIV infection.^20^ Because there is no cure for AIDS, prevention remains the only sure way for combating the disease.^21^ Adolescents are often victims of the pandemic, because of reasons such as limited awareness of HIV and/or AIDS or inadequate access to HIV prevention and treatment services.^22^ The study could thus add new insights in terms of the effect of such community-based initiatives and preventative campaigns among adolescents in at-risk rural settings.

## Methods

### Study population and sample

A qualitative approach was followed and a descriptive case study design employed to conduct the study. The target population comprised all adolescents whose parents had died of HIV and/or AIDS-related illnesses, who reside in a rural community in the Eastern Cape Province of South Africa. This village represents at-risk rural communities in South Africa and is characterised by high levels of poverty, HIV and/or AIDS prevalence and vulnerable children – some because of being orphaned and some as a result of parents not residing with their children. A sample of 16 children (10 boys and 6 girls), aged 11–24 years, were included in the study.

### Selection of participants

Purposive sampling was used to select the participants. Community members played a crucial role in the initial identification of adolescents who could potentially provide rich information on the HIV and/or AIDS awareness among youth in the specific community. As community members knew the children of the community, they were viewed as important access point and key role-players in the sampling process.

The selection criteria that applied stipulated that participants (1) had to be adolescents from a rural community in the Eastern Cape, (2) had to be between the age of 11 and 24 years, (3) had lost one or both parents because of HIV and/or AIDS, (4) who were available for participation in the project, and (5) whose caregivers provided informed consent for their participation. Adolescents whose parents had died of any disease or event other than HIV and/or AIDS and children who fell outside the stipulated age range were thus excluded from the study. The participants’ demographics are summarised in Table 1.

**TABLE 1:** Participants’ demographics in the study.

Orphanhood status	Male participants	Female participants
Maternal orphan	3	4
Paternal orphan	2	2
Double orphan	5	0

### Ethical considerations

Approval to conduct the study was first sought from the University of Fort Hare, who approved the research proposal and proposed instruments for data collection and documentation. Informal meetings with leading people in the study community were held to discuss the research project, proposed time frames, ways of sampling and participation. Introduction of both the researcher and assistant researchers was done during these meetings in order to identify and select suitable participants and obtain permission to conduct the research in the seven subdivisions of the village. The Headman subsequently provided written permission to the research team.

After obtaining permission from the relevant authority figures, written consent was obtained from the caregivers and parents of the adolescent participants. Only children with signed consent forms participated in the study.

The necessary measures were taken to ensure confidentiality, anonymity, autonomy and respect for privacy.^23^ In obtaining individual informed consent, participants were informed that the study would include sensitive questions and they were reminded that their participation was voluntary and that they had the right to withdraw from the study at any time without penalty. As the study progressed, caution was taken to identify any child experiencing discomfort or potential emotional harm, yet no such incidences occurred. The research team was, however, prepared to refer any such cases for professional support if these were to occur.

### Data collection and documentation

Semi-structured individual interviews were conducted with the 16 participants, focusing on their experiences and awareness of the HIV and/or AIDS pandemic and related issues. Interviews lasted an average of 45 minutes each, with no interview being longer than an hour. All interviews were guided by a pre-formulated interview guide.

Interviews were conducted in IsiXhosa – the home language of the participants. All interviews were voice recorded and transcribed verbatim following the data collection sessions. Two Xhosa-speaking MA Psychology students from the Psychology Department of the University of Fort Hare conducted the interviews and translated the transcripts into English. An independent researcher who served as project supervisor, whose origin is also Xhosa, confirmed the back translation as a true reflection of what was said by the participants. Observations and interviews were furthermore captured by means of field notes.

## Data analysis

Inductive thematic analysis was used, which resulted in the identification of three main themes. Atlas.ti7 was employed as software programme assisting in the identification of codes and related quotations and memo purposes. Atlas.ti7 made the data analysis process easier and more effective. For interpretation of the data, an interpretivist paradigm was adopted.^24^

As stated, all interviews were transcribed in preparation of the analysis. After completing the transcriptions, data were organised according to a set of questions and responses. The thematic analysis was further used to uncover themes and trends that are similar to ideas and concepts which formed the main themes of the study.

## Results of the study

Following inductive thematic analysis, three main themes were identified. The first theme relates to participants’ awareness of HIV and/or AIDS, and the second theme relates to the disclosure of their parents’ status and the third theme relates to their own experiences when their parents passed on.

### HIV and/or AIDS awareness

Results revealed that the majority of the participants were aware of HIV and/or AIDS and aspects related to the pandemic. Awareness centred around three topics, namely that HIV and/or AIDS can but do not necessarily have to result in death, that it is an infectious disease and that several sources of information could be accessed in the community.

Firstly, participants indicated an awareness that HIV and/or AIDS can ‘kill’ and has resulted in the death of many people. This idea is captured in the following contribution:

‘HIV kills, it killed my mother.’ (P06, female, 14 years old)

Participants, however, also seemed aware that HIV and/or AIDS does not necessarily have to result in death and that living a positive life is a possibility if one accepts one’s status, as captured in the following extract from the data:

‘When one accepts his or her HIV status, one can live with it.’ (P10, male, 21 years old)

Secondly, several participants articulated that AIDS is an infectious disease and can be transmitted. They stated:

‘I heard people saying you must not touch the other person’s blood or you will be infected.’ (P02, male, 13 years old)

‘It is a disease that is sexually transmitted and through body fluids for example blood and semen.’ (P07, female, 20 years old)

‘I know about HIV. One gets it through infected blood and sex.’ (P08, male, 20 years old)

In this regard, participants also referred to protection and using contraceptives. They shared the following views:

‘One gets HIV and/or AIDS by sleeping with someone without a condom and he or she will die.’ (P01, male, 18 years old)

‘I know what HIV and/or AIDS is, one gets it when the boyfriend has it and has sex without protection.’ (P11, female, 19 years old)

Next, participants identified the sources where they had learnt about HIV and/or AIDS, indicating their schools and the local clinic. They commented:

‘I learnt about HIV from my teacher at school.’ (P02, male, 13 years old)

‘I learnt about HIV from school through class discussion and my friends.’ (P08, male, 20 years old)

‘At school in the Life Orientation subject and Nurses visited our school and taught us about HIV.’ (P11, female, 19 years old)

‘I learnt it from school and clinic when I tested for it.’ (P03 [HIV-positive], female, 23 years old)

Some participants also shared how experience with their own family members served as a source of knowledge to them:

‘I saw it from my father.’ (P05, female, 17 years old)

‘I learned about HIV from my grandmother.’ (P06, female, 14 years old)

Despite the majority of the participants indicating awareness of HIV and/or AIDS, a few participants revealed not knowing enough:

‘I do not know much, all I know I used to have it but not anymore. I was given a treatment that healed me.’ (P04, female, 12 years old)

Quite surprisingly, two participants apparently had no knowledge on the pandemic, saying:

‘Nothing, I have never heard about it.’ (P12, male, 12 years old)

‘I don’t know anything. I have not paid attention to it as far as information is concerned.’ (P15, male, 16 years old)

### Disclosure of parents’ HIV and/or AIDS status

As part of the study, it was investigated whether participants had knowledge on the HIV status of their parents and how they came to learn about it. Participants expressed different views on this, with some of them merely becoming aware that their parents were sick:

‘My mother got sick but I did not know with what, her feet were swollen.’ (P02, male, 13 years old)

‘No, I did not know, he just said to me he was having chest pains.’ (P04, female, 12 years old)

Unsurprisingly, some of the parents reportedly did not want their children to know about their HIV status, with the participants only realising this after their parents had passed away. The following examples apply:

‘She was hiding her status from me but her boyfriend told me after the funeral.’ (P03, female, 23 years old)

‘No, I only knew after my father died.’ (P08, male, 20 years old)

Some of the participating adolescents indicated that they read their parents’ medical cards and then realised their HIV and/or AIDS status. One participant reported:

‘I knew because I saw my father’s clinic card and he developed a rash and sores. I had a picture of him sick in hospital.’ (P05, female, 17 years old)

In some other cases, grandparents informed the participants, for example:

‘Yes my grandmother told me about my mother’s status.’ (P06, female, 14 years old)

‘I knew that she was HIV-positive. My grandmother told me when she was seriously ill.’ (P07, female, 20 years old)

In some cases, however, participants reported that they did not have any knowledge of their parents’ HIV and/or AIDS status. The following excerpts provide examples:

‘I did not have any idea whether they had HIV and/or AIDS or not. Both my parents were sick but I did not know what was wrong with them.’(P15, male, 16 years old)

‘I did not know I was only two years when they died.’ (P16, male, 12 years old)

### Experiences of parents’ deaths

The majority of the participants associated their own recollections of their parents’ deaths with a distinct awareness of their parents constantly being sick and in pain. They shared the following experiences:

‘My mother was always sickly until she died. I was not staying with her so I could not know how she looked like.’ (P03, female, 23 years old)

‘He was in pain all the time. Very weak. I was consistently worried about him.’ (P04, female, 12 years old)

‘I remember him being sick until he died.’ (P05, female, 17 years old)

When sharing their observations of their parents being chronically ill, participants also indicated concern on their side both prior to and following the death of their parents, as captured in the following contribution:

‘My father was always sick and going to the clinic and taking pills. I was really worried before he died. When he died, life was hard, we could not get everything we wanted by the time we wanted it.’ (P08, male, 20 years old)

In addition to their parents being sick, participants reportedly noticed physical signs and symptoms associated with AIDS and AIDS-related deaths. The namely mentioned weight loss and noticeable pimples on their parents’ bodies, reporting as follows:

‘My mother was epileptic. She became sick with unknown sickness and she lost her weight.’ (P06, female, 14 years old)

‘She lost so much weight she had pimples all over her body and she was coughing severely.’ (P07, female, 20 years old)

‘My father had shingils and was unable to do anything for himself. My mother looked fine because she was doing everything for us. There were no visible symptoms.’ (P15, male, 16 years old)

A few participants could, however, not recall anything about their parents’ deaths primarily because they were still young at the time of death. One participant for example said:

‘I did not remember well because they died while I was very young.’ (P01, male, 18 years old)

## Discussion

In terms of HIV and/or AIDS awareness, the adolescents in this at-risk rural community showed some insight into the fact that HIV and/or AIDS is an infectious transmittable disease that can cause death but can also be prevented. A study by Oyo-Ita et al.,^25^ which aimed to establish the impact of HIV and/or AIDS awareness programmes among Secondary School adolescents in Nigeria, indicates similar findings, with the majority (90%) of the participants in that study knowing that HIV and/or AIDS can be transmitted through sexual intercourse. Surprisingly though, some participants indicated that they were not aware of HIV and/or AIDS or specific information related to the pandemic. This apparent lack of awareness is most probably rather an indication of denial among some adolescents in the community, to possible reasons such as them not having dealt with the death of their parents, or fearing their own HIV and/or AIDS status. This is a mere hypothesis that requires further investigation; however, against the background of HIV and/or AIDS information being frequently reported in the mass media,^26^ the chances of adolescents having no knowledge on the topic seem rather slim. In their study, Oyo-Ita et al.^25^ indicate television and the radio as primary sources of information on HIV and/or AIDS. Even though these authors propose follow-up, the information sessions by, for example, teachers and/or parents, basic information is seemingly conveyed via mass media, also in far-off remote and rural communities. Detailed information seems to be limited to only a percentage of children, as indicated by Oyo-Ita et al.^25^ who found that only 22.6% of adolescents receive information from teachers and 2.2% receive information from parents.

The current study established that the majority of the participants in this study possess accurate knowledge on HIV and/or AIDS prevention and infection. These findings are supported by Singh and Jain^20^ who also indicate that a large number of adolescents know that HIV and/or AIDS is preventable, by, for example, abstaining from sexual relationships, or having a single, uninfected and faithful partner.^20^ Other studies similarly indicate that the majority of adolescents possess the correct knowledge about HIV and/or AIDS.^27^ In a recent study by Mwamwenda^21^ that assessed the level of HIV and/or AIDS knowledge of high school adolescents in Kenyait, it was concluded that, even though the level of HIV and/or AIDS knowledge was high, some misconceptions existed, which would justify the continued promotion of public education regarding HIV and/or AIDS. The study furthermore showed that public education does produce dividends in the prevention of HIV and/or AIDS transmission and the spread of the pandemic.^21^

The finding that some of the participants in the current study (however limited) showed a lack of awareness of HIV and/or AIDS emphasises the need for re-enforced school HIV and/or AIDS education. In addition, adolescents – especially orphaned adolescents living in poor communities – should receive life skills training. By equipping vulnerable adolescents with basic skills such as problem-solving skills, conflict management techniques, self-awareness and self-protection, goal setting and career planning, they may be empowered to protect themselves from being exploited or harmed. In this way, the danger of transactional sexual exploitation and abuse may be combated. At present, South African children receive life skill training as part of the national curriculum; however, the need for practical skills application remains.

Even though teachers play a pivotal role in imparting knowledge, the use of multi-pronged methods, such as films, group discussions, dramas, puppet shows and role plays, can also be incorporated with success, as proven by Harvey et al.^28^ It is, however, also important to provide the necessary resources to sustain such programmes or interventions and to obtain more evidence on the effect on behaviour, by, for example, determining changes in behaviour and HIV and/or AIDS incidence following such interventions.^28^

The current study furthermore indicates that parents who died of AIDS or HIV and/or AIDS-related illnesses tend not to disclose their status to their children despite adolescents becoming aware of the status of their parents through different sources. This finding is supported by Naswa and Marfatia,^3^ who found that disclosure of HIV and/or AIDS is associated with multifaceted challenges. As adolescence implies emotional vulnerability, the way in which children from this age group will respond to a disease status can never be predicted. Key considerations mentioned by the WHO^29^ relate to stigma being a major barrier to parents or caregivers disclosing their HIV and/or AIDS status to their children, fearing that children may be stigmatised. Furthermore, the manner in which disclosure takes place varies from culture to culture and from place to place, depending on available resources and caregivers’ desires and concerns.^29^

The decision to disclose to children involves weighing the pros and cons of disclosing against one another. Delaney et al.^30^ assert that this decision is often based on the child’s perceived ability to handle the information without being psychologically harmed. Another alternative relates to people potentially fearing that disclosure could disrupt their current or future relationships.^31^ Disclosure may thus be avoided if people fear abandonment, rejection, discrimination or upsetting family members. As such, parent’s disclosure of his or her HIV and/or AIDS status to children may potentially affect the child’s adjustment or the parent–child relationship. Even if children are not informed of the status of their parents, they may, however, intuitively know that something is wrong with their parents or even notice them taking medication. The current study confirms this possibility as some children became aware of their parents’ HIV and/or AIDS status when seeing their parents’ medical clinic cards or through observation of physical symptoms such as their parents’ loss of weight and the presence of pimples.

Despite Murphy et al.^32^ observing that parents often choose not to disclose to their children in order to avoid negative responses, research shows (Dane cited in Qiao et al.^33^ Dolly and Dillon cited in Armistead et al.^34^ and Murphy et al.^35^) that there are many benefits when children are aware of their parents’ HIV status. Even though participating adolescents in the current study were not directly asked about this, it appeared as if they wished that their parents had made them aware of their illness before they passed away. Murphy et al.^32^ confirm that children may initially respond negatively to disclosure, but that parents and healthcare providers can guide children to accept such a diagnosis by validating their emotional responses, clarifying any misperceptions about the disease and providing emotional support.

It should be noted, however, that the findings of this study do not imply that parents who had died of HIV and/or AIDS-related illnesses never disclosed their HIV and/or AIDS status to anybody, as this was not explored. In their study, Ramdas et al.^36^ found that, according to patients’ records, most patients (90.7%, *N* = 54) disclosed their HIV and/or AIDS status. Women prefer to disclose to relatives (82.1%, *>N*> = 28) rather than to partners (28.6%, *N* = 28). A few participating adolescents in the current study also revealed that relatives (grandmothers) and partners of their parents informed them about the HIV and/or AIDS status of their parents after they had died.

## Conclusion

This study shows that the majority of adolescents in at-risk rural community in South Africa are knowledgeable about HIV and/or AIDS infection and prevention, thereby confirming related studies in this field. Even though preventative and informative-focused interventions and campaigns therefore seem to reach far-off rural communities, whether through mass media or other avenues, it is also argued that continued educational campaigns are required, for example, to also guide parents in terms of discussions on HIV and/or AIDS with their children, including discussions of their own status. Recommendations can furthermore include information on disclosure of HIV and/or AIDS status in ways and by people that can potentially reduce the possibility of stigma and discrimination.

Community health workers, nurses, social workers and counsellors can fulfil supporting roles in enhancing parents’ and caregivers’ skills to communicate about HIV and/or AIDS with children. More intervention is furthermore needed to address stigma and discrimination against people living with HIV and/or AIDS and their families. This need for change has been an ongoing priority in research on HIV and/or AIDS^37^ and remains to require urgent attention.
